# Supplementary material for: Complexome profiling of the Chlamydomonas psb28 mutant reveals TEF5 as an early PSII assembly factor
Source: Plant Cell. 2025 Mar 17;37(6):koaf055. doi: 10.1093/plcell/koaf055 (PMC12164590; doi:10.1093/plcell/koaf055)
Supplement: koaf055_Supplementary_Data [file koaf055_supplementary_data.zip › psb28_interactive.zip!/plots/intensity/qualityPlots/peptides/TOM5_Cre06.g276001.t1.1.html]

### Cre06.g276001.t1.1

Trivial name: TOM5  
SeqCoverage [median]: 20.40  
Mass: 5.98 kDa  
  
 

| Sequence | MissedCleavage | Mass [Da] | wtIntensity | muIntensity | Score |
| --- | --- | --- | --- | --- | --- |
| ASNDLLTSAQK | 0 | 1146.59 | 7952.60 | 11831.64 | 270.36 |
|  |  |  | 7952.6 | 11831.6 |  |
